# Leukemic Stem Cell: A Mini-Review on Clinical Perspectives

**DOI:** 10.3389/fonc.2022.931050

**Published:** 2022-06-24

**Authors:** Igor Valentim Barreto, Flávia Melo Cunha de Pinho Pessoa, Caio Bezerra Machado, Laudreísa da Costa Pantoja, Rodrigo Monteiro Ribeiro, Germison Silva Lopes, Maria Elisabete Amaral de Moraes, Manoel Odorico de Moraes Filho, Lucas Eduardo Botelho de Souza, Rommel Mário Rodriguez Burbano, André Salim Khayat, Caroline Aquino Moreira-Nunes

**Affiliations:** ^1^ Department of Medicine, Pharmacogenetics Laboratory, Drug Research and Development Center (NPDM), Federal University of Ceará, Fortaleza, Brazil; ^2^ Department of Pediatrics, Octávio Lobo Children’s Hospital, Belém, Brazil; ^3^ Department of Biological Sciences, Oncology Research Center, Federal University of Pará, Belém, Brazil; ^4^ Department of Hematology, Fortaleza General Hospital (HGF), Fortaleza, Brazil; ^5^ Department of Hematology, César Cals General Hospital, Fortaleza, Brazil; ^6^ Center for Cell-based Therapy, Regional Blood Center of Ribeirão Preto, University of São Paulo, São Paulo, Brazil; ^7^ Ceará State University, Northeast Biotechnology Network (RENORBIO), Fortaleza, Brazil

**Keywords:** hematopoietic stem cells, leukemia stem cell, molecular biomarkers, clinical relapse, drug resistance

## Abstract

Hematopoietic stem cells (HSCs) are known for their ability to proliferate and self-renew, thus being responsible for sustaining the hematopoietic system and residing in the bone marrow (BM). Leukemic stem cells (LSCs) are recognized by their stemness features such as drug resistance, self-renewal, and undifferentiated state. LSCs are also present in BM, being found in only 0.1%, approximately. This makes their identification and even their differentiation difficult since, despite the mutations, they are cells that still have many similarities with HSCs. Although the common characteristics, LSCs are heterogeneous cells and have different phenotypic characteristics, genetic mutations, and metabolic alterations. This whole set of alterations enables the cell to initiate the process of carcinogenesis, in addition to conferring drug resistance and providing relapses. The study of LSCs has been evolving and its application can help patients, where through its count as a biomarker, it can indicate a prognostic factor and reveal treatment results. The selection of a target to LSC therapy is fundamental. Ideally, the target chosen should be highly expressed by LSCs, highly selective, absence of expression on other cells, in particular HSC, and preferentially expressed by high numbers of patients. In view of the large number of similarities between LSCs and HSCs, it is not surprising that current treatment approaches are limited. In this mini review we seek to describe the immunophenotypic characteristics and mechanisms of resistance presented by LSCs, also approaching possible alternatives for the treatment of patients.

## Introduction

Hematopoietic stem cells (HSCs) are located in the bone marrow (BM) and are responsible for sustaining and regenerating the hematological system. It is estimated that in a human organism, 1x10^6^ blood cells are produced every second. This feature comes from the ability of self-renewal together with a high proliferative rate and pluripotency of these cells. It is also worth mentioning the ability to resist apoptosis, necrosis and genotoxicity produced by reactive oxygen species (ROS) that HSCs have (1–9).

Most of the time, the HSCs are quiescent, at G0 phase of the cell cycle, depending on glucose to carry out their metabolic activities. However, these cells, when receiving the stimuli through severe situations, can quickly enter the cell cycle through activation of genetic factors. It begins with a positive control carried out in part by mTORC1 under the action of CDK6, which in the G0 phase is in low expression or accompanied by inhibitors such as p57 or p18 (8, 10–13).

HSCs enters the cell cycle, and therefore, their metabolic activities start to have mitochondrial oxidative phosphorylation as a source of energy due to the increase in energy demand. This metabolic alteration consequently triggers a series of proteins, such as histone and DNA modifying enzymes, which are fundamental for the epigenetic changes carried out by the modulation of key transcription factor activity. After their activation, HSCs generate multipotent progenitors that are then committed to a cell lineage and gradually differentiate until they become mature and specialized cells (8, 10–12, 14–16).

Due to aging, HSCs lose their regenerative ability and may undergo a process called age-related clonal hematopoiesis (ARCH). In this process, mutations acquired over time continue to be transmitted to their successors, giving rise to cells with mutations. Patients with ARCH are more likely to develop leukemias, however not all cells in this process will be related to the leukemic process. It is known that the presence of certain mutations is related to the severity factor of this cell, as mutations in *TP53* and *U2AF1* genes are associated with pre-leukemic stem cells, and mutations in DNMT3A and TET2 genes have a lower risk regarding transformation of malignancy (12, 17–19).

In this study, we investigated clinical trials in extended literature that focused their efforts on the identification of LSCs in different types of leukemia and we discussed their clinical outcome and the perspectives of new therapies.

## Pre-Leukemic Stem Cell and Leukemic Stem Cell

The constant accumulation of mutations occurring in HSCs due to ARCH or other agents can stimulate the transformation of HSC into a pre-leukemic tumor cell (pre-LSCs). Although mutations are present in these cells, it is still possible for them to continue to give rise to healthy cells. However, pre-LSCs continue to accumulate mutations for years as well as significant clonal expansion until, after a long period, this cell acquires malignant characteristics, becoming a leukemic stem cell (LSC) (20–23). Despite the similarities between these two types of cells, it is still possible to make differentiations, mainly genetic differentiations, where it is observed that pre-LSCs do not have mutations associated with leukemia (12, 24, 25).

**Figure 1:**
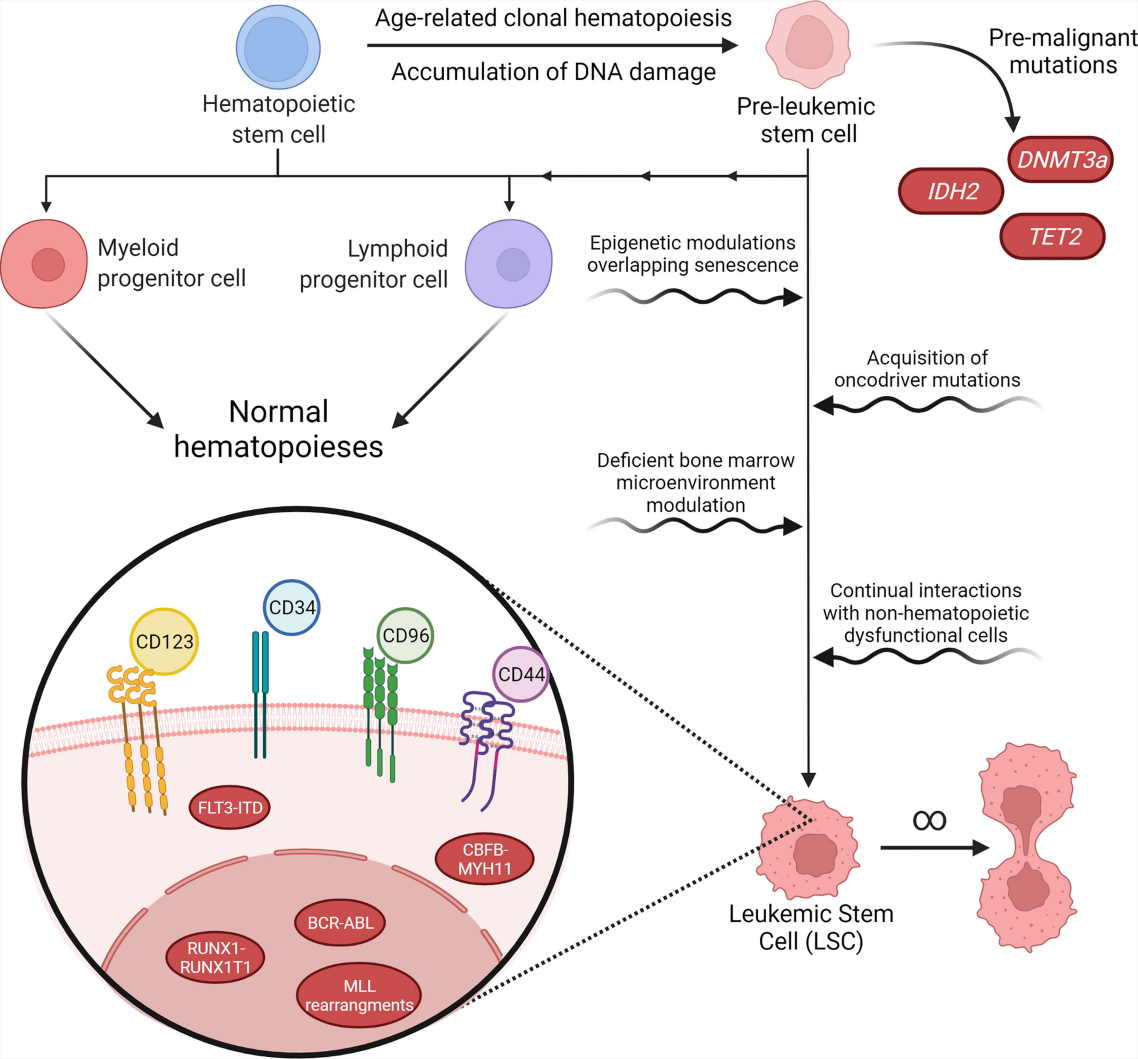
Pathways of malignancy in hematopoiesis and its characteristics. Aging and exposure to hazardous environmental agents lead to accumulation of DNA damage and mutations in hematological precursor cells, inducing a pre-leukemic stem cell (pre-LSC) phenotype. Pre-LSCs acquire proliferation advantages over normal hematopoietic stem cells (HSCs) due to mutations in genes such as DNA methyltransferase 3 alpha (*DNMT3a)*, but still retain their capacity to promote normal hematopoiesis. However, further malignant characteristics acquired over the years may tip these cells into a proper leukemic stage. The transformation of pre-LSCs may happen through cell-specific processes, such as epigenetic modulation or new acquired mutations, or through interactions between these cells and their microenvironment, through changes in the normal growth and survival signaling pathways or due to interactions with dysfunctional stromal or mesenchymal cells that are also present in the bone marrow. After malignancy onset, leukemic stem cells (LSC) may present a variety of karyotype rearrangements, such *as BCR-ABL* or *FLT3-ITD*, that determine their malignant characteristics and tend to present immunophenotyping profiles that still resemble normal HSCs, such as CD34^+^38^-^, while also overexpressing a cohort of cell-surface antigens that are highly variable between patients and even among different cell populations in the same patient.

Mutations that occur in pre-LSCs are related to epigenetic genes that are responsible for histone modification, DNA methylation and chromatin looping. In pre-LSCs mutations can be found in *AML1, ASXL1, CTCF, DNMT3A, E2H2, FOXO1, IDH1, IDH2, IKZF1, JAK2, NPM1, MED12, SMC1A, STAT5B, TET2* and *WT1*. These mutations alone are incapable of inducing leukemia and appear as precursor events to late mutations that transform pre-LSCs into LSCs, which is shown in **Figure 1**. Therefore, the genetic alterations present in LSCs are related to proliferation and active signaling (26–33).

LSCs are recognized by their stemness features such as drug resistance, self-renewal, and undifferentiated state. They were initially pointed out by Lapidot and colleagues in 1994 (34). This rare population of resistant cells is believed to be at the origin of leukemia relapses. Their quiescent state and their self-renewal capacity makes it possible to leukemia repopulating cells, despite their low frequency (35–39).

LSCs are present at low levels in BM, being found in only 0.1%, approximately. This makes their identification and even their differentiation difficult since, despite the mutations, they are cells that still have many similarities with HSCs (20). Although the common characteristics, LSCs are heterogeneous cells and have different phenotypic characteristics, genetic mutations, and metabolic alterations. This whole set of alterations enables the cell to initiate the process of carcinogenesis, in addition to conferring drug resistance and inducing relapses (36, 37, 40).

During tumor progression, cancer cells continuously acquire genetic changes, and the fittest, most proliferative cells are selected for giving rise to distinct tumor subclones, which is known as clonal hematopoiesis (CH) (41–44). The clonal hematopoiesis of indeterminate potential (CHIP) refers to the presence of at least one driver mutation in hematopoietic cells of peripheral blood, without hematological malignancy. It is associated with increased risk of cancers, particularly in myeloid neoplasms, and chronic inflammatory diseases. The phenomenon of CHIP becomes very common in the population of people aged ≥80 years. That is explained by the accumulation of somatic mutations in HSCs, which occurs in an age-dependent manner (45, 46).

Clones evolve through the interaction of selectively advantageous ‘driver’ lesions, selectively neutral ‘passenger’ lesions and deleterious lesions. Driver lesions or mutations are the mutations that increase fitness and confer a clonal growth advantage. The neutral mutations, also known as passenger mutations, are accumulated in these cells but do not confer any fitness advantage. Neutral evolution of these passenger mutations can also shape clonal evolution, notably by a phenomenon called genetic drift, in which the allele frequencies of a mutation change over time. In addition, when both driver and passenger mutations occur in the same cell, the passenger mutations increase their allele frequency with the driver mutations, which is a phenomenon called hitchhiking that also participate in clonal evolution (47, 48).

The recognition of the important role of clonal hematopoiesis and clonal evolution in tumor initiation, disease progression and relapse have profound implications for the diagnosis and treatment of these malignancies. Additionally, the advent of new technologies may facilitate the definition of the molecular determinants and underlying mechanisms of clonal evolution in leukemia, which could provide targeted, individualized therapies for leukemia patients (49, 50).

## Characterization of Leukemic Stem Cells

A major challenge in studying LSCs is identifying a possible unique cell surface antigen phenotype, from which it would be possible to develop a targeted and much more specific treatment than the treatments currently used. Furthermore, due to the heterogeneity of the different types of leukemias, there is a lot of variation in the antigens found on the surface of these cells, becoming even more difficult to identify a specific marker that is not expressed in normal cells, or has a different expression pattern, density, or distribution (51–54).

Some common stem cell’s markers are CD34, CD117 and HLA-DR, which expressions predict lower rate of complete remission (CR). In addition, the principal surface antigens of the myeloid lineage are CD13, CD33, CD14, CD15 and CD11b. Mostly, the expression of these markers have not yet showed any prognostic significance or are associated with a poorer outcome, such as reduction of CR, period of remission and survival. Although, cells that expresses CD15 usually presents a higher CR rate. Another marker found in myeloblasts is CD56, which expression is also reported as a poorer prognostic factor (55)

As an example, we can point out that normal HSC constitutively expresses CD34^+^ and CD38^-^ antigens, in addition to others such as Thy-1^+^, c-kit^+^ and IL-3Rα. Much of the LSC population immunophenotypically resemble certain normal hematopoietic progenitor populations by also expressing CD34^+^ and CD38^-^ besides others surface markers (56–58)

Despite these difficulties, a great number of cell surface markers have been identified that are upregulated on CD34^+^CD38^-^ LSCs compared with normal CD34^+^CD38^-^ HSPCs, for example, it has been revealed that CD90 and CD117 are deficient in acute myeloid leukemia (AML) LSCs, while CD123, TIM3, CD47, CD96, CLL-1, and IL-1 receptor accessory protein (IL1RAP), G protein-coupled receptor 56 (GPR56), CD93, CD44 and CD99 are highly expressed in AML LSCs. Targeting these surface markers might be a promising strategy for eradicating AML LSCs (35, 56, 59–62).

Some studies addressing chronic myeloid leukemia (CML) LSCs demonstrated that differentially expressed antigens include CD25, CD26, IL-1RAP, which is associated with the activation of NF-kβ and AKT signaling pathways, increasing proliferation of CML LSCs. In addition, the overexpression of the antigen CD25 is reported to reduce proliferation capacity of CML LSCs. Some data suggest CD25 and IL-1RAP expression are unique to LSCs of this type of leukemia (54, 63, 64).

The presence of LSCs is related to the rates of complete remission (CR) and general survival (OS) of patients, besides that, depending on the remaining amount, they may predispose to relapse of patients with leukemias (65). The identification of certain surface markers and molecular changes in these cells may influence the prognosis of patients, but the results of studies are still somewhat controversial. Bradstock et al. (66) pointed out that patients who expressed CD9, CD14 and CD2 in their CSLs had lower CR rates. The CD9, CD10 and CD11b markers were associated with lower OS rates, and CD11b was also related with a shorter duration of CR.

A study by Béné et al. (67) demonstrated that the expression of CD10, CD14 and CD15 was associated with lower survival rates. Nomdedeu et al. (68) found that patients who expressed CD34, CD45, CD117 and CD123 had worse prognoses with lower OS. Other studies, in turn, pointed out the markers CD2, CD7, CD11b, CD22, CD133, CD135, CD262 and CD120a as markers that confer worse prognosis to patients (69, 70).

The relationship between the molecular alterations observed in patients and their prognosis is also somewhat controversial among studies. Nomdedeu et al. (68) demonstrated that the *FLT3-ITD* mutation had a significant influence on OS, where patients affected by this alteration had lower rates compared to those who did not have the mutation (17.9 vs 41 months). On the other, Béné et al. (67) did not find a difference in survival between patients with and without molecular changes. However, other articles report the relationship of *FLT3-ITD, MLL-PTD, RUNX1-RUNX1T1* and *CBFB-MYH11* mutations with a poor outcome (71, 72).

In addition, studies demonstrated a correlation between white blood cells (WBC) count and poorer prognosis. Patients with a higher WBC count were less likely to achieve CR and presented a shorter survival rate (67, 73). Besides, a higher platelet count was associated with a longer survival time (67). These general findings corroborate with data found in other articles (74–77).

## Presence of LSCS and Relapse

Even with the course of the disease, it is possible to observe that pre-LSCs and LSCs continue to evolve throughout the process, in addition to the fact that treatment often fails to reach these cells. LSCs play a key role from development to disease relapse. Thus, its analysis and quantification can be of great importance as a prognostic factor for patients. Such processes can also assist in choosing a more targeted treatment. The identification of these cells can be performed through immunophenotyping, where it is possible to differentiate HSCs from LSCs. From this differentiation, counting methods are performed (78–80).

Recent studies point to two classifications of relapse related LSCs: the first classification is known as LSCs of committed relapse origin, where these cells are most like the diagnostic cell type and were able to evolve similarly to the diagnostic dominant clone. The second classification is the LSCs of primitive relapse origin, which are rare cells at the diagnosis of the disease and do not usually form blasts. However, these cells may show greater resistance to treatment and later clonal evolutions, causing the patient to relapse, which usually continues with the increase in the amount of LSCs and with greater heterogeneity (81, 82).

Studies reveals that LSCs levels correspond to the clinical and laboratory characteristics of the patient. Due to their insufficient morphological and biochemical characterization, LSCs cannot be reliably measured in patient samples. However, as a consequence of the finite capacity of the joined stem cell niche, HSCs can act as a biomarker for LSCs numbers and help identify patients with an adverse clinical outcome (74, 83, 84).

This is noticeable through the LSCs and the HSCs count, considering that the BM may have a limited number of cells. Therefore, LSCs and HSCs compete for niches, their values ​​being inversely proportional, that is, the more LSCs the less HSCs. Therefore, patients who had a low LSCs load also had lower blast, platelet, and leukocyte counts. In addition to clinical features, the cells count can represent how it might respond molecularly. This is important when evaluating the chosen treatment. Therefore, lower levels of LSCs are associated with a better molecular response to treatment (85, 86).

Still on the quantification of LSCs, its functionality also applies to the assessment of measurable residual disease (MRD) and is considered an effective biomarker for predicting relapses. So, in addition to being used in the diagnosis to choose the treatment, the immunophenotyping test can be used in the post-treatment phase to evaluate its effectiveness and predict the patient’s survival. High values ​​of LSCs would be associated with worse survival and low efficacy. This analysis is then performed using molecular methodologies such as quantitative real-time polymerase chain reaction (RT-qPCR) or next-generation sequencing (NGS), and flow cytometry. It is important to remember that all these methodologies for prognostic analysis and MRD are still being carried out in studies and better clarification and standardization are needed for clinical application (83, 84, 87, 88).

## Insights Into Clinical Investigations

**Table 1** is comprised of clinical trials from the past 10 years that aimed to identify biomarkers specific to LSCs that could serve as targets for targeted therapies or could be used as prognostic factors (89–98). Most of the reported studies aim at AML treatment since LSC presence and complexity is an established risk factor for disease severity (51, 56).

**Table 1:** Studies of the past 10 years indicating biomarkers for stem cells and “stemness” properties in leukemia and the respective prognostic relevance after treatment.

Leukemia Subtype	Alterations Correlated with LSC Phenotype	Treatment Protocols	Clinical outcomes	Reference
AML	Pre-leukemic phenotype of CD34^+^CD13^+^CD33^+^ and increased expression of CD123 and CD117	Intensive and non-intensive induction regimens	Association of pre-leukemic phenotype with persistent clonal hematopoiesis and increased mutation burden	(89)
AML; MDS	Expression of CXCR4/CXCL12	Azacitidine plus DSTAT	ORR of 27% among evaluable patients and major hematologic improvements	(90)
AML	Upregulation of CD70/CD27 interaction	Protocols of cusatuzumab plus azacitidine administration	Strong reduction of LSC viability and proliferation *in vitro*; 100% ORR in 12 analyzed patients with 44% of evaluable patients achieving MRD negativity	(91)
AML	Lower expression of miR-204 increasing the expression of CD34 cell marker	Standard protocols of induction chemotherapy	Low expression of miR-204 is associated with poorer OS and DFS	(92)
AML	High CD123 expression	Standard protocols of induction chemotherapy	Overexpression of CD123 is associated with poor OS and induction therapy failure	(93)
AML	Expression of CD25, CD96 and CD123	Standard protocols of induction chemotherapy	Expression of multiple surface markers is associated with worse OS, PFS and response to chemotherapy	(94)
AML	Presence of CD34^+^CD123^+^ and CD33^+^CD123^+^ cells	CZBG combined with standard chemotherapy regimens	Reduction of CD34^+^CD123^+^ cells in the bone marrow after treatment	(95)
AML	Expression of CD44, CD123 and CD184	Variable protocols of cytotoxic chemotherapy	Increased LSC population is correlated with inability to achieve CR	(96)
CML	*BCR-ABL* translocation in CD34^+^lin^-^ cells	Nilotinib 300mg twice a day	No *BCR-ABL* rearrangement was observed in analyzed CD34^+^lin^-^ cells in the bone marrow at 12 months of treatment	(97)
CML	*BCR-ABL* translocation in CD34^+^CD38^-^ cells	Dasatinib 100mg a day or imatinib 400mg a day	Rapid decrease in LSC and LPC populations after therapy initiation	(98)

LSC, Leukemic Stem Cell; AML, Acute Myeloid Leukemia; NR, Not Reported; MDS, Myelodysplastic Syndrome; DSTAT, Dociparstat Sodium; ORR, Overall Response Rate; MRD, Minimal Residual Disease; OS, Overall Survival; DFS, Disease-free Survival; PFS, Progression-free Survival; CZBG, Compound Zhebei Granule; CR, Complete Response; CML, Chronic Myeloid Leukemia.

Studies utilizing standard chemotherapy protocols and induction therapies confirm that identified LSC markers, mainly transmembrane antigens such as CD34 and CD123, correlate with a worse patient prognosis. Increased mutation burden, lower response rates, inability to achieve CR, worst response to chemotherapy and higher incidence of relapse are some of the reported factors associated with increased presence of LSCs (89, 92–94, 96).

Although resistant to most treatment strategies, the use of novel agents targeting specifically LSCs molecular pathways combined with standard treatment protocols showed some promising results for AML patients (90, 91, 95). Huselton et al. (90) combined dociparstat sodium (DSTAT), a drug capable of inhibiting CXCR4/CXCL12 cell adhesion molecules, with hypomethylating agent (HMA) azacitidine in an attempt to disrupt bone marrow niches where LSCs remain in a quiescent state and was able to achieve CR in patients who were previously unresponsive to treatments with HMA alone.

Riether et al. (91) identified WNT pathway as being hyperactivated in AML LSCs due to increased expression and interaction of CD70/CD27 molecules. *In vitro*, the use of monoclonal antibodies targeting CD70 in combination with HMAs was demonstrated to have an additive effect in inhibiting LSC growth since the use of HMAs seem to increase LSCs dependency on CD70/CD27 pathway and concurrent CD70 inhibition was able to further reduce LSCs burden when compared to monotherapies of either agent. In previously untreated AML patients, protocols combining cusatuzumab plus azacitidine induced responses in all treated patients and transcriptome analysis after treatment revealed increased expression of genes involved in pathways of inflammation, differentiation and apoptosis (91).

Lastly, Wang et al. (95) utilized compound zhebei granule (CZBG), a herbal concoction with oncologic uses in traditional Chinese medicine, that acts through mechanism such as apoptosis induction and inhibition of resistance-related drug efflux proteins, combined with standard chemotherapy to treat AML patients and a significant decrease in CD34^+^CD123^+^ cells was observed in bone marrow niches. CZBG was also demonstrated to increase response rates in AML patients in combination with chemotherapy when compared to chemotherapy alone and to be able to reduce LSCs markers in tumor cell xenografts when combined with doxorubicin treatment (95, 99, 100).

In CML patients, *in vitro* studies indicate *BCR-ABL* tyrosine kinase inhibitors (TKIs) to have no efficacy over LSCs and leukemic progenitor cells (LPCs) and, while imatinib initial response rates are overwhelmingly positive, disease recurrence is usually the standard for patients after therapy discontinuation due to remaining Philadelphia-positive (Ph^+^) CD34^+^ cells (101, 102). The use of next-generation TKIs, however, seem to be more effective in reducing stem and progenitor cells in CML than imatinib and may point towards a choice for more intensive treatment options in accordance with increased LSCs and LPCs burden (103).

Pungolino et al. (97) utilized nilotinib, a second-generation BCR-ABL inhibitor, to treat newly diagnosed CML patients and observed a rapid decrease in CD34^+^lin^-^Ph^+^ cells in the bone marrow, with total clearance of the analyzed samples at 12 months of treatment. Mustjoki et al. (98) compared imatinib and dasatinib efficiency at decreasing stem and progenitor cell burden and, while both treatments had similar results at LSC inhibition, dasatinib showed increased activity over LPCs levels at 3 months analysis.

## Treatment Perspectives

Most conventional treatments for leukemias seek to eradicate blasts, reaching cells that are in their active cycle. However, LSCs are usually in a quiescent state or protected through their molecular resistance mechanisms causing this cell to resist therapy, leading to patient relapse (104–111). One of the major known factors that confer drug resistance is the overexpression of drug efflux pumps, such as the ATP-binding cassette (ABC) transporter family proteins by CSCs. Moreover, drug efflux increase is often combined with the upregulation of enzymes involved in the metabolism of anticancer agents. Therefore, enzymes and efflux transporters expressed by LSCs appear to be crucial not only for their proliferation, but also for their resistance to clinical treatments (108, 112–115).

The selection of a target to LSCs therapy is fundamental. Ideally, the target chosen should be highly expressed by LSCs, highly selective, absence of expression on other cells, in particular HSCs, and preferentially expressed by a high number of patients. In view of the large number of similarities between LSCs and HSCs, it is not surprising that current treatment approaches are limited (20, 81, 116).

Currently, different treatment methodologies have been tested and addressed, such as the use of binding antibodies associated with different toxins to form a specific delivery vehicle for LSCs. Examples of such therapies are the use of Gemtuzumab Ozogamicin for the treatment of AML, a compound that uses an antigen against CD33, associated with a cytotoxic agent; and the inhibition of the SIRP1-α interaction with CD47 that activates innate immunity increasing the death of LSCs. In addition, CD244, CD123, LLC1 or TIM3 targets are also studied and demonstrate antileukemic efficacy in AML patients. However, one of the main difficulties in the treatment of LSCs is due to the low proliferation rate, which makes it difficult to identify the cell to start the therapy (62, 81, 117–119).

Several new strategies are under development to eliminating LSCs, which may result in a better patient response. Many of the studies are associated with the use of TKIs, which to improve their effectiveness can be combined with other agents. TNF-α inhibitors combined with TKIs, for example, have shown positive results in the elimination of LSCs. Blocking of IL-1 signaling may also be a combination with TKIs, as well as blocking of signal pathways such as Wnt/β-catenin, Hedgehog, MAPK/MNK1/2, mTOR, PTEN, PP2A, Alox5 and JAK/STAT. The action of HIF-1 inhibitors associated with TKIs has been shown to reduce the survival and growth of cells in CML in murine models. The *HIF-1* deletion has also been tested in *in vivo* and *in vitro* models and has been shown to inhibit CML proliferation, both without serious effects on HSCs (120–122).

Activations and gene dissections can also be used, as in the case of p53 activation and *EZH2* deletion. Both technologies demonstrate promising results that enhance the eradication of LSCs when combined with TKIs (123, 124). The combination of TKIs with cytarabine was also performed, demonstrating good results in AML patients. Despite the large number of tests involving TKIs, medications and methodologies have also been developing as the case of Bortezomibe. Its function is based on decreasing the expression of CDK6, an important agent in the proliferation of LSCs (120, 125, 126).

Undoubtedly, one of the greatest difficulties in eliminating LSCs is their resistance mechanisms. As a result, studies have specialized in finding drugs and technologies that help to overcome the resistance present in LSCs. In this scenario, research diverges in different areas such as transport proteins and signaling pathways, taking as an example Notch, Hedgehog, and Wnt/β-catenin that are describes also as responsible for drug resistance (104, 127–129). It is also worth mentioning studies focused on the epithelial-mesenchymal transition (EMT), histone acetylation, hypoxia and the BM niche (104, 130, 131).

The tumor microenvironment (TME) creates a niche for itself that influences not only the proliferation and differentiation of LSCs but also the response to drugs. A key factor that modulates the microenvironment and drug resistance is hypoxia, which signaling contributes to chemoresistance of CSCs by increasing the expression of ABC transporters and Aldehyde dehydrogenases, a family of intracellular enzymes, which can be used as molecular markers to identify normal stem cells (NSCs) and CSCs (104, 128, 132–134). An example was demonstrated by Giuntoli et al. (134), when CML cells were grown in low oxygen concentrations and became resistant to Imatinib.

The EMT process is already known in solid tumors and has recently been explored in hematological neoplasms as well as its treatment possibilities (104, 127, 134, 135). Thus, one of the ways is to look for drugs that can act on genes such as *TRPS1, ETS2* and *LSP*, known to belong to this process in AML (136, 137). Competition for the BM niche between LSCs and HSCs has also become a therapeutic target, transforming the environment in a more favorable way for HSCs or increasing their hematopoietic reserve. E-selectin inhibition is an example, being able to promote the displacement of HSCs and LSCs. Regarding hypoxia, the use of hypoxia-activated prodrugs such as TH-302 has already been shown to reduce the population of LSCs in an AML model (138, 139).

In addition to those already mentioned, the combination of Venetoclax with Azacitidine demonstrates potential for the treatment of LSCs in AML, as it suppresses *OXPHOS*. Regarding gene deletion, it was observed that *FOXO1* deletion through genetic or pharmacological pathways is able to inhibit the proliferation of malignant cells, which is present in LSCs and pre-LSCs, becoming a potential target. Studies aimed at the use of microRNAs mimics were also carried out and have potential, such as miR-15a/16-1 acting as a tumor suppressor acting negatively on the *WT1* gene (28, 81, 140, 141).

## Conclusion

Foremost is important to better define the molecular and cellular biologic features of normal HSCs and LSCs, for improve the identification of possible therapeutic targets to eradicate the LSCs, that are responsible for treatment resistance and clinical relapse for most patients. It is necessary to carry out studies that correlate the quantification and immunophenotypic characterization of LSCs with clinical data and prognosis presented by patients, regarding the significance of this information pointed in the studies here presented, and due to the lack of this type of study in the literature.

## Author Contributions

IB, FP and CM wrote the manuscript. All authors contributed to the manuscript and were involved in revisions and proof-reading. All authors contributed to the article and approved the submitted version.

## Funding

This study was supported by Brazilian funding agencies: Coordination for the Improvement of Higher Education Personnel (CAPES; to CM), National Council of Technological and Scientific Development (CNPq grant number 404213/2021-9 to CM-N; and Productivity in Research PQ Scholarships to MM-F, MM, RB and AK), Cearense Foundation of Scientific and Technological Support (FUNCAP grant number P20-0171-00078.01.00/20 to FP, MM and CM-N) and we also thank PROPESP/UFPA for publication payment.

## Conflict of Interest

The authors declare that the research was conducted in the absence of any commercial or financial relationships that could be construed as a potential conflict of interest.

## Publisher’s Note

All claims expressed in this article are solely those of the authors and do not necessarily represent those of their affiliated organizations, or those of the publisher, the editors and the reviewers. Any product that may be evaluated in this article, or claim that may be made by its manufacturer, is not guaranteed or endorsed by the publisher.
